# Mutational Analysis of Merkel Cell Carcinoma

**DOI:** 10.3390/cancers6042116

**Published:** 2014-10-17

**Authors:** Derek J. Erstad, James C. Cusack

**Affiliations:** 1 Department of Surgery, Massachusetts General Hospital, 55 Fruit Street, Boston, MA 02114, USA; E-Mail: derstad@partners.org; 2 Division of Surgical Oncology, Harvard Medical School, Massachusetts General Hospital, 55 Fruit Street, Boston, MA 02114, USA

**Keywords:** merkel cell carcinoma, merkel cell polyomavirus, tumor suppressor, oncogene, prognostic algorithm, mutational analysis

## Abstract

Merkel cell carcinoma (MCC) is an aggressive cutaneous neuroendocrine malignancy that is associated with a poor prognosis. The pathogenesis of MCC is not well understood, and despite a recent plethora of mutational analyses, we have yet to find a set of signature mutations implicated in the majority of cases. Mutations, including TP53, Retinoblastoma and PIK3CA, have been documented in subsets of patients. Other mechanisms are also likely at play, including infection with the Merkel cell polyomavirus in a subset of patients, dysregulated immune surveillance, epigenetic alterations, aberrant protein expression, posttranslational modifications and microRNAs. In this review, we summarize what is known about MCC genetic mutations and chromosomal abnormalities, and their clinical significance. We also examine aberrant protein function and microRNA expression, and discuss the therapeutic and prognostic implications of these findings. Multiple clinical trials designed to selectively target overexpressed oncogenes in MCC are currently underway, though most are still in early phases. As we accumulate more molecular data on MCC, we will be better able to understand its pathogenic mechanisms, develop libraries of targeted therapies, and define molecular prognostic signatures to enhance our clinicopathologic knowledge.

## 1. Introduction

Merkel Cell Carcinoma (MCC) is a rare and aggressive cutaneous neuroendocrine tumor. MCC is most often found in elderly Caucasians approximately 60–80 years old, with an annual incidence in the United States of approximately three cases per million persons per year, though this number has nearly tripled in the last 20 years with an aging populace, increased UV exposure and greater frequency of immunosuppression in the population [1]. MCC can be up to 13 times more frequent in immunosuppressed patient populations including those with HIV, organ transplants and certain hematologic cancers including multiple myeloma, non-Hodgkin’s lymphoma and chronic lymphocytic leukemia [2,3]. Although the relationship between immunosuppression and MCC is not entirely understood, the discovery of the Merkel cell polyomavirus (MCPyV) infection in up to 80% of cases offers a potential mechanism for malignant transformation, and may provide more insight in this regard [4]. The mechanisms of oncogenesis underlying MCPyV-negative MCC are less well understood, but are thought to involve somatic mutations in tumor suppressors including RB1 and TP53, as well epigenetic alterations resulting in aberrant expression and activity of oncogenes [5,6]. The extent of disease at presentation is a strong predictor of survival, ranging from a 70% 10-year-survival rate in patients with isolated local disease, to 20% or less in patients with distant spread [7]. Male sex, tumor size, clinical nodal status, metastatic dissemination, lymphovascular invasion, high mitotic index and small cell morphology are associated with poor prognosis [8,9].

MCC is most frequently found on the head and neck, followed by the upper extremities, lower extremities and trunk. Less than one percent of cases are diagnosed in the parotid and submandibular glands, nasal cavity, and lymph nodes. Tumors present as firm, flesh-colored (often with red or blue hues), painless nodules that are fast growing and tend to metastasize early to lymph nodes and other distant organs. Histopathologically, hematoxylin and eosin staining of MCC presents as round blue tumor cells, indicative of large basophilic nuclei with minimal cytoplasm, located in the dermis or subcutaneous tissue. They may have a trabecular pattern, scant eosinophilic cytoplasmic rims, multiple nucleoli and paranuclear staining of cytokeratin-20 (CK-20) in a dot-like pattern. Normal merkel cells are located within the stratum basale and rete ridges of epidermis, as well as in mucosa [10,11]. They are of ectodermal origin and function in light touch with slow adapting somatosensory afferent fibers. There has been limited debate that MCC may not be derived from merkel cells, but rather pluripotent stems cells within the skin [12].

MCC has historically been difficult to study and treat due to limited epidemiologic data, variable response to treatment and outcomes, and lack of associated genetic mutations for targeted therapy. Approximately half of the available articles on MCC have been published within the last five years, of which few are prospective randomized clinical trials. Although consensus guidelines for management of MCC exist, there are still unanswered fundamental clinical questions regarding the best use of surgery, chemotherapy and radiation for this condition.

## 2. The Role of Mutational Analysis in MCC

MCC response to treatment modalities and prognosis is variable, and clinical and histologic characteristics have limited utility to predict outcome. Underlying the perplexing natural history of MCC are unique differences in chromosomal abnormalities, genetic mutations, expression profiles and epigenetic controls of individual tumors that are still poorly understood. Recently, Merkel cell polyomavirus (MCPyV) has been found in up to 80% of MCCs in most reported series and is associated with improved outcome compared to virus negative tumors [13]. Better understanding of MCC at the molecular level will provide much needed insight regarding prognosis, prediction of response to aggressive surgical excision and chemoradiation, and the development of targeted therapy.

## 3. Merkel Cell Polyomavirus

A key distinction in the mutational analysis of MCC is MCPyV status. Discovered in 2008, this is one of the 13 known polyomaviruses that naturally infect humans, though it is the only human polyomavirus thought to be involved in tumorigenesis [13]. Up to 60%–80% of the normal population will test positive for MCPyV infection, whereas the incidence of infection among MCC patients is approximately 80%–90% [14]. Among those infected, anti-VP1 antigen titer is significantly higher in MCC patients compared to the normal population [15]. It is not understood why the infection rate or antigen titer are higher in MCC patients, or what determines oncogenic transformation in infected patients, though immunocompromise likely plays a role. The incidence of MCC is 15-fold higher among immunocompromised patients, and up to 30-fold higher in patients with certain liquid malignancies [16,17]. However, it has not been validated to our knowledge that the incidence of MCPyV positive MCC is higher in the immunocompromised population than in non-immunocompromised patients. MCPyV status of tumors is independent of patient age, whereas MCPyV status among the normal population is age dependent [18].

The MCPyV virus integrates its DNA into tumor cells in a clonal pattern, preceding tumor proliferation. There are two relevant viral proteins: large T antigen (LT-Ag) and small T antigen (ST-Ag), which have been implicated in oncogenesis through multiple mechanisms. ST-Ag contains an N-terminal J domain similar to LT-Ag, but is otherwise structurally unique. ST-Ag was shown to inhibit proteasomal degradation of LT-Ag and other SCF(bw37) ubiquitin ligase targets, including c-Myc and cyclin E, contributing to oncogenesis [19]. ST-Ag has also been found to transform rodent fibroblasts via preservation of hyperphosphorylation of 4E-BP1 at S65 resulting in dysregulated cap-dependent translation that was resistant to mTORC1 and two inhibitors [20]. 4E-BP1 functions via inhibition of eukaryotic translation initiation factor 4E (eIF4E), which is a limiting component of the multisubunit complex that recruits 40S ribosomal subunits to the 5' end of mRNAs for translation initiation. Knockdown of ST-Ag alone results in growth arrest of MCC cell lines [21].

LT-Ag has four putative regions: an N-terminal J-domain, RB1 and DNA binding motifs, and a C-terminal helicase domain. The viral genomes recovered from MCC tissue contain mutations affecting the helicase and DNA binding regions that selectively inhibit LT-Ag ability to support viral replication, thereby preventing lytic viral replication that could be lethal to a cancer cell, while still maintaining its Rb-binding capacity and cell cycle dysregulating function. The helicase region has been shown to promote growth inhibitory functions, likely through induction of DNA damage response kinases, which may partly explain why signature truncation of this region is found in MCC [22]. Perhaps, the most frequently documented oncogenic function of LT-Ag is its RB1 binding capacity. MCC-derived truncated LT-Ag binds RB1 with high affinity, partially relocalizing the protein to the cytoplasm and suppressing its anti-neoplastic function [23]. This Rb-sequestering function was shown to be essential to virus positive MCC proliferation both in *in vitro* and xenograft models [24]. LT-Ag also downregulates expression of TLR9, a key receptor in the host innate immune response that senses viral or bacterial dsDNA, thereby liberating infected cells from host immune surveillance. LT-Ag achieves this affect via inhibition of C/EPBβ binding at the TLR9 promoter [25]. Unlike other polyomaviruses, full length and truncated MCPyV lack TP53-binding capacity [26]. Most MCCs are TP53 wild type and increased TP53 expression is associated with worse prognosis, therefore large and small T antigens may affect TP53 function indirectly.

MCPyV positive tumors are more commonly found in females and are associated with lower stage and better prognosis, including longer overall and disease free survival [4,27,28,29]. Histologically, MCPyV positive tumors have been found to have less nuclear polymorphism and cytoplasmic content, consistent with their less sinister course. A higher viral copy number per tumor cell is associated with improved survival in complete remission [4]. The association between viral infection and prognosis is poorly understood, though may in part be related to immune response. The presence of tumor infiltrating cytotoxic T cells (CD8+) is independently associated with improved prognosis, and MCPyV positive tumors have greater numbers of intra- and peritumoral CD3+ and CD8+ T cells [30,31]. As expected, on transcriptome analysis, virus positive tumors transcribe significantly more immune response genes [18]. Alternatively, it may be that virus-negative tumors have more frequent and aggressive somatic mutations. Though there is limited data to support this hypothesis, it has been shown that deletion of RB1 and mutations in TP53 are more common in virus negative MCCs [32].

Targeted immunotherapy towards T cell antigens and their downstream targets may be promising for virus positive tumors. Although up to 80% of the general population will harbor antibodies to MCPyV capsid proteins, titers are significantly higher in MCC patients, who also uniquely generate antibodies to T antigens. Anti-T antigen titer may be used as a biomarker for disease regression or recurrence in a subset of patients. Monoclonal antibodies, vaccines and adoptive cellular approaches targeting T antigens and other MCC-specific tumor antigens are being studied as potential therapeutic modalities [33].

## 4. Mutations in Tyrosine Kinase Signaling: KIT, PDGFRA, PIK3CA, AKT and PTEN

PDGFRA and KIT (CD117) are transmembrane receptor tyrosine kinases associated with certain cancers including melanoma and acute myeloid leukemia, and activating mutations in both genes have been associated with tumorigenesis [34]. MCCs have been found to express both proteins, warranting investigation as therapeutic targets [35].

Andea *et al.* evaluated KIT expression in 30 MCC tumors, which was elevated in 67% of cases and was associated with a trend towards worse prognosis. Point mutations were found in exons 17 and 18, and introns 16 and 17, though no activating mutations were identified [36]. In a similar study, 23 cases of MCC were evaluated for KIT and PDGFRA expression and mutational status. 65% of tumors expressed CD117 and 95% expressed PDGFRA. In 12 of 18 samples, a single nucleotide polymorphism (SNP) in KIT exon 11 resulted in an E583K amino acid change, which has previously been described as an inactivating mutation in piebaldism, and is unlikely related to tumorigenesis. A SNP with silent effect in exon 18 of PDGFRA was found in eight of 18 samples [37]. Kartha *et al.* evaluated 14 primary and 18 metastatic MCC tumors for KIT and PDGFRA expression and mutation. KIT and co-expression of its ligand, SCF, was found in 16% of cases, whereas co-expression of PDGFA and PDGFRA was observed in 81% of cases. Silent mutations were observed in exon 17 of KIT and exons 10, 12 and 18 of PDGFRA, but activating mutations were not found [38]. Based on these findings, activating mutations in KIT and PDGFRA receptor tyrosine kinases are unlikely drivers of MCC tumorigenesis. Although these proteins may play a role in MCC cellular proliferation and survival, the mechanism underlying their aberrant expression and activity is poorly understood.

Nardi *et al.* sequenced select mutational hotspots of 60 MCC tumors and found three (5%) TTP53 point mutations and six (10%) PIK3CA activating point mutations [39]. PIK3CA has been implicated in multiple cancers including: liver (36%), breast (26%), colon (25%), urothelial (13%), ovarian (9%), gastric (7%), brain (6%), and lung cancer (2%) as well as leukaemia (1%) [40,41]. It serves as an intracellular tyrosine kinase that activates AKT downstream to stimulate cell cycle progression via mTOR, cellular proliferation via NF-κb, and inhibition of apoptosis via deactivation of tumor suppressors including TP53, p21, p27 and GSK3b. Five of the six PIK3CA mutations observed were within the helical domain of the p110a subunit, which is mutated in a wide variety of skin cancers [42]. These mutations were exclusively found in men, and associated tumors included a mix of primary and recurrent disease, had a normal distribution pattern, were significantly larger in size (>2 cm) with worse stage, had more necrosis and pleomorphic spindle cell features, and all were MCPyV negative except for one case. However, the authors were unable to correlate these findings to worse prognosis, likely due to limited power. They tested multiple PIK3CA inhibitors and were able to inhibit phosphorylation and activation of AKT in multiple MCC cell lines and induce apoptosis in one line with both ZST474, a specific phosphoinositide 3-kinase (PI3K) inhibitor, and NVP-BEZ235, a dual PI3K/mTOR inhibitor. Although no mutations were observed in the AKT gene, it was shown that a subset of MCC samples display high AKT activity in the setting of wild type PIK3CA, suggesting upstream activation either through an unknown oncogene or disinhibition from mutated tumor suppressor, specifically PTEN [39].

Hafner *et al.* also evaluated the PIK3CA pathway in MCC and found 2/46 (4%) MCC had PIK3CA mutations and none in AKT. However, activating phosphorylation of AKT was found in 88% of MCCs, which was uncorrelated with MCPyV status, and cells were sensitive to the PIK3CA inhibitor LY-294002 [43]. Based on these data, upstream or epigenetic aberrations are likely driving the pathway given the lack of intrinsic mutations.

PTEN (phosphatase and tensin homologue) is a tumor suppressor implicated in many cancer types, which functions by inactivating AKT via dephosphorylation. Chromosomal analysis of 21 MCC samples showed hemizygous mutations in nine (43%) samples of the10q23 region of Ch10, where PTEN is located. However, homozygous deletions or point mutations of the remaining allele were quite rare, suggesting alternate mechanisms of PTEN inactivation or the involvement of other tumor suppressors in MCC [44].

More work is needed to elucidate the mechanisms of constitutive PIK3CA/AKT activation in MCC given the relative paucity of pathway mutations, and to better characterize the function of these genetic aberrations in oncogenesis and prognosis. Tyrosine kinase inhibitors may still play an important chemotherapeutic role, and there are currently multiple ongoing clinical trials (Table 1). Most are still in early phases and incomplete. The only completed to date trial is a phase II study of imatinib that showed no benefit in advanced MCC patients [45].

**Table 1:** Clinical trials for tyrosine kinase inhibitors in Merkel Cell Carcinoma (MCC).

Generic Name	Trade/Code Name	Mechanism of Action	Trials in other Cancers	MCC Trial Phase	Trial Status	Additional
Pazopanib [46]	Votrient	Multi-targeted tyrosine kinase inhibitor	Renal cell, soft tissue sarcoma, lung, ovarian, breast, prostate, neuroendocrine, thyroid, cervical, cholangiocarcinoma, germ cell, urothelial and fallopian tube cancers	Phase 2	Recruiting	
Cabozantinib [47]	Cometriq	Targeted inhibitor of c-Met and VEGFR2	Thyroid, melanoma, breast, pancreatic, prostate, brain, bladder and ovarian cancers	Phase 2	Recruiting	
Nelfinavir [48]	Viracept		Pancreatic, brain, cervical, head and neck, rectal, soft tissue sarcoma, and non-small cell lung cancers	Phase 1	Unknown	
Cixutumumab [49]	IMC-A12	Monoclonal antibody targeting IGF-1R	Esophageal, soft tissue sarcoma, lung, liver, prostate, melanoma, breast, colorectal and thymoma cancers	Phase 1	Ongoing, not recruiting	In combination with Everolimus
Everolimus [49,50]	Afinitor	Inhibitor of mTOR	Breast, brain, pancreatic, liver, colorectal, lung, head and neck, fallopian tube, gastric, thyroid, prostate, endometrial, renal cell, and cervical cancers	Phase 1 *, Phase 1 **	Ongoing, not recruiting *, Ongoing, not recruiting **	Separate trials in combination with Cixutumumab and Vatalanib
Vatalanib [50]	PTK787	Inhibitor of VEGF-1 and 2, PDGFRβ and KIT	Hematologic, GIST, pancreatic, brain, colorectal, prostate, breast, melanoma, lung and mesothelioma cancers	Phase 1	Ongoing, not recruiting	In combination with Everolimus
Temsirolimus [51]	Torisel	Inhibitor of mTOR	Thyroid, prostate, breast, liver, head and neck, endometrial, ovarian, neuroendocrine, gastric, cervical, pancreatic, renal, lung, colorectal, esophageal and brain cancers	Phase 1	Ongoing, not recruiting	In combination with Vinorelbine
Imatinib [52]	Gleevec	Inhibitor of BCR-ABL	Hematologic, GIST, ovarian, breast, head and neck, lung, colorectal, thyroid, testicular, prostate, renal, gastric, brain, melanoma, pancreatic and sarcoma cancers	Phase 2	Completed	No observed benefit

VEGFR2, Vascular Endothelial Growth Factor Receptor 2; IGF-R1, Insulin Growth Factor-1 Receptor; mTOR, Mammalian Target of Rapamycin; VEGF-1/2, Vascular Endothelial Growth Factor 1/2; PDGFRβ, Platelet Derived Growth Factor Receptor Beta; BCR-ABL, Breakpoint Cluster Region-Abelson Murine Leukemia gene. * Everolimus and Cixutumumab combination therapy trial; ** Everolimus and Vatalanib combination therapy trial.

## 5. Mutations in Tumor Suppressors: TP53 and RB1

Mutations in the TP53 tumor suppressor have rarely been found in MCC, ranging from 0%–28% in most studies, the majority of which represent SNPs or silent mutations with unknown or no clinical significance [53,54,55,56]. Of note, Waltari *et al.* 2011 analyzed 87 MCC tumors and found no TP53 mutations, though TP53 protein expression was detected in 22.8% of samples, and was associated with MCPyV negative status and worse disease specific (*p* = 0.021) and overall survival (*p* = 0.46) [55]. Lassacher *et al.* evaluated 21 MCC tumors for mutations in tumor suppressors and oncogenes commonly seen in skin cancers. They found three mutations in TP53 (14%) and one mutation in p16INK4a, though no mutations in Ha-Ras, N-Ras, KIT or p14ARF. However, inactivating p14ARF promoter methylation was present in eight of 19 analyzable samples (42%), suggesting that epigenetic tumor suppressor silencing may play a role in MCC oncogenesis [56]. In contrast, the tumor suppressor p73, a structural homologue of TP53, was mutated in four of 15 MCC samples, with unclear significance [57]. Based on these studies, TP53 inactivating mutations unlikely contribute to MCC oncogenesis.

Prior work has validated that TP53 expression is a marker for poor prognosis in multiple tumor types [58]. Normal functioning TP53 is typically undetectable at the protein level due to its short half-life and rapid turnover. Although in certain cases missense mutations in TP53 can prevent its degradation and tumor suppressive function, the majority of MCC samples studied to date have had wild type TP53, and therefore other mechanisms are likely at play. In this regard, the mouse double minute 2 homolog (MDM2) protein may be of therapeutic significance. This protein forms a complex with TP53 in the cytoplasm, preventing the tumor suppressor from binding its responsive element and initiating anti-proliferative and DNA repair mechanisms. Due to this sequestration, TP53 accumulates and remains nonfunctional [59]. This process has been described in sarcoma, where it was shown that MDM2 was amplified in one third of 47 samples, and was specifically associated with detectable expression of TP53 [60]. Houben *et al.* looking specifically at MCC, studied whether T antigens contributed to TP53 stabilization and found that viral knockdown did not lead to resumed TP53 function. However, they found that inhibition of MDM2 by the compound Nutlin-3a did induce TP53 transcriptional activation, resulting in tumor cell apoptosis in five of seven lines with wild type TP53 [61].

Retinoblastoma (Rb) inactivation is thought to play an important role in the pathogenesis of MCC. In MCPyV-positive cancers, sustained tumor growth is contingent on the presence of a functional large T antigen with intact RB1 binding domain to sequester and inactivate the tumor suppressor, which may serve as an important therapeutic target for the subset of MCPyV-positive cancers [23,24]. RB1 is also frequently downregulated in virus negative tumors, though the mechanisms of inactivation are still under investigation. Leonard *et al.* evaluated 24 MCC samples for hemizygous deletions, and found that 75% of tumors contained deletions in chromosome 13 near the RB1 locus [62]. A separate study showed that virus negative tumors had increased genomic instability compared to virus positive MCCs with higher rates of deletion in the RB1 locus that correlated with decreased RB1 detection by immunohistochemistry. Additionally, they found RB1 promoter hypermethylation in all tumor samples, irrespective of MCPyV status and RB1 expression [5]. In addition to chromosomal deletion, a higher frequency of nonsense truncating mutations in the RB1 gene has been shown in MCPyV-negative tumors [63].

## 6. Chromosomal Abnormalities

Chromosomal aberrations can potentially provide insights into the pathogenesis of MCC, reveal specific gene targets, and serve as a diagnostic resource. Initial forays into chromosomal analysis in MCC utilized comparative genomic hybridization (CGH) to define copy number abnormalities, but lacked the resolution to isolate specific gene candidates. Frequently amplified regions have been found on chromosomes 1, 5, 6, 8 and 20, and frequent losses on chromosomes 13 and 4 (Table 2) [6,64,65]. Chromosomal alterations are associated with larger tumors at higher risk for metastatic dissemination [64]. However, most studies lack evidence for high-level amplifications [66]. The advent of microarrays has greatly improved the resolution of hybridization, and can provide copy number information at the single gene level. Using array-CGH technology, Paulson *et al.* evaluated 23 MCC samples, and similarly found that tumors frequently carried additional copy regions of chromosomes 1, 3q, 5p, and 6 and lost chromosomes 3p, 4, 5q, 7, 10 and 1. MCPyV positive tumors had fewer genetic alterations. Three chromosomal regions were of interest, including a deletion of 5q12–21 found in 26% of tumors, a deletion of 13q14–21 also found in 26% of tumors that contains the RB1 tumor suppressor, and amplification at 1p34 present in 39%, which contains the L-Myc (MYCL1) oncogene [67].

**Table 2:** Chromosomal abnormalities in MCC.

Chromosome	Deletion/Amplification
1	Amplification of 1p34 in 9/23 (39%) tumor samples, contains L-Myc [67].
	Deletion of 1p32–33, 1p35 and 1p36 in 16/24 (73%) of MCC tumor samples, 1p36.33 contains p73 tumor suppressor [57].
	Amplification of 1q11q31 in 32% of 19 primary MCC tumors analyzed, high-level amplification of 1q22q24 in 5% of samples [64].
	Deletion of 1p35–36 in 7/10 (70%) of MCC samples [68].
	Deletion of 1p arm in 3/3 (100%) of MCC samples [69].
3	34 tumors samples from 24 patients revealed frequent loss for chromosomes 3p (46%), 5q (21%), 8p (21%), 10 (33%), 11q (17%), 13q (33%) and 17p (25%), and gains were seen for chromosomes 1 (63%), 3q (33%), 5p (38%), 8q (38%), 19 (63%), and X (41%) [70].
	18/25 (69%) of tumor samples demonstrated 3p deletions ranging from 3p13–p21.1 [71].
5	Amplification of 5p in 32% and high-level amplification of 5p in 5% of 19 MCC samples [64].
	Deletion of 5q12–21 in 26% cases of 23 tumor samples [67].
6	Amplification of 6p in 8/19 cases (42%), most common 6pterqter [64].
	Trisomy in 8/17 cases (47%) [72].
	Trisomy in 2/4 lymph nodes samples and 6/10 primary tumor samples [73].
	Trisomy documented in a single patient case report of disease recurrence [74].
7	Case report document deletion of the long arm with break point at 7q31, as well as trisomy of chromosomes 6 and 11 [75].
8	Trisomy documented in a single patient case report of disease recurrence [74].
	Amplification of 8q21–q22 and loss of 4p15-pter [6].
10	Deletion of 10q23 in 9/21 (43%) cases, containing the PTEN locus [44].
13	Deletion of 13q13q31 (21%), 4q (16%), and 16q (11%) in 19 MCC samples [64].
	Deletion of 13q14–21 in 26% of 23 tumor samples [67].
	Deletion of 13p in 18/24 75% cases, most commonly deleted region was mapped close to the RB1 susceptibility gene 13p14.3 [62].
22	Case report documenting deletion of 22p in 100% and 22q in 85% of MCC cells from a patient sample [76].

PTEN, Phosphatase and tensin homolog.

## 7. MicroRNAs

MicroRNAs (miRNAs) are non-coding RNA sequences approximately 18–22 bases in length that silence translation of complementary messenger RNA transcripts, thereby regulating post-transcriptional gene expression. Discovered within the last 20 years, miRNAs have been shown to play critical roles in multiple biologic processes, and they are often deregulated in cancers. Certain miRNAs have been directly linked to oncogenesis, and provide potential diagnostic, therapeutic and prognostic value.

Xie *et al.* evaluated miRNA expression patterns of MCC, and found distinct expression profiles based on MCPyV status. Specifically, miR-203, miR-30a-3p, miR-769-5p, miR-34a, miR-30a-5p, and miR-375 were significantly different between the two groups. They also identified multiple miRNAs associated with decreased survival and metastases, including: miR-150, mi-146, miR-630, miR-483-5p, and miR-142-3p. However, only miR-150 was statistically significant, and may potentially serve as a useful prognostic marker [77].

MiR-203 suppresses multiple targets involved in oncogenesis, and is downregulated in certain cancers [78,79,80,81]. In MCC, miR-203 has been shown to suppress expression of survivin, a highly conserved member of the inhibitor of apoptosis (IAP) family that is overexpressed and contributes to tumorigenesis [82]. Xie *et al.* showed that miR-203 overexpression resulted in decreased transcript and protein detection of the survivin gene, which was associated with increased cell cycle arrest, though in MCPyV negative cells only. In virus positive tumors, LT-Ag is thought to inhibit survivin expression via sequestration of the RB1 tumor suppressor, and RNA silencing of LT-Ag was able to restore susceptibility to miR-203 overexpression. Thus, survivin expression appears to be differentially regulated by miR-203 and LT-Ag in virus negative and positive MCCs, respectively [77]. Recently, YM-155, a direct survivin inhibitor, has been shown be cytotoxic to MCPyV positive MCC cells at nanomolar concentrations in mouse xenografts, improving survival, and therefore may serve as a potential therapeutic target for MCC [83]. In MCPyV negative cells, miR-203 delivery may provide an alternative novel therapeutic target.

Ning *et al.* evaluated the miRNome in MCC and found significant upregulation of miR-502-3p, miR-9, miR-7, miR-340, miR-182, miR-190b, miR-873, and miR-183 relative to non-MCC cutaneous tumors and normal skin controls. They found downregulation of miR-3170, miR-125b, and miR-374c [84]. miR-125b downregulation in breast and hepatocellular carcinomas is associated with disinhibition of cellular proliferation and anti-apoptotic programs, and overexpression may restore regulatory mechanisms [85]. In contrast, in melanoma, miR-182 expression is associated with tumor proliferation and invasion, likely via suppression of the FOXO3 tumor suppressor [86]. The role of under- and overexpression of miRNAs in MCC is still poorly understood, though may provide a novel library of therapeutic targets.

## 8. Negative Mutational Findings

MCC pathogenesis has been difficult to characterize given the abundance of negative mutational studies (Table 3). To date, several highlights have been reported: mutations in TP53 ranging from approximately 5%–28% in most series, variable deletion of the RB1 locus, and more recently, a novel study reporting a 10% frequency of mutation of PIK3CA in 60 MCC samples [5,39,56]. Aberrant expression and activity of both tumor suppressors and oncogenes have been frequently documented in MCC, yet the paucity of associated mutations suggests that this cancer may lack a defining profile such as the BCR-ABL mutation in chronic myelogenous leukemia. Rather, oncogenesis is likely predicated on poorly understood dysregulated processes including epigenetic programs, post-transcriptional gene regulation, and post-translational modifications.

**Table 3:** Mutational analyses with negative findings in MCC.

Negative Study	Description
p14ARF, p16INK4, H-Ras, K-Ras, N-Ras, KIT	1/20 (5%) p16INK4 mutations, no mutations in any of the other genes; hypermethylation of p14ARF suggesting downregulation of the tumor suppressor [56].
p73 and TP53	Missense mutation in p73 of unclear significance in 15 samples. One TP53 nonsense mutation [57].
PTEN	Hemizygous mutations in 9/21 MCC samples, though remaining allele unmutated. Epigenetic silencing of remaining allele is possible though yet to be shown [44].
PDGFA	Expression detected in 25/31 (81%) of cases, though no activating mutations [38].
c-KIT	Expressed in 8/9 (89%) of cases, though no activating mutations [87].
Wnt	Elevated β-catenin in only 1/12 (8%) cases, no mutations. Similarly no mutations in APC [88]. Lill *et al.* 2013 found no increased expression of β-catenin or cyclin D in MCC samples [89].
BRAF	No mutations in exon 15 (commonly mutated region in melanoma) in 15 samples tested [90]. No. V600E mutations, which is found in 43% of melanomas, in 46 MCC samples [91].
MAPK Pathway	High expression of Raf Kinase Inhibitor Protein (RKIP), which deactivates the pathway. Expression though lack of phosphorylated activation of ERK in 42/44 (95%) cases, representing lack of activation [91].
ALK	Expressed in 26/32 (81%) of MCC samples, no rearrangement or other cytogenetic abnormality of the locus identified [92].
HRAS, KRAS, NRAS, BRAF, cKIT	No mutations in exons 1 and 2 of all genes studied in 6 MCC cell lines [6].
RON and MSP	No mutations, though transcription of both genes in 9/14 MCC samples and no controls [93].
Notch-1	Tumor suppressor downregulated in lung and gastrointestinal neuroendocrine tumors, found to be expressed in 30/31 (97%) of MCC samples, thus unlikely to play a role in oncogenesis. Mutational status no evaluated [94].
APC, BRAF, β-catenin, EGFR, FLT3, JAK2, cKIT, KRas, NRas, Notch-1, PTEN	No mutations in hotspots of these genes in 60 MCC samples [39].

p14ARF, p14 Alternate Reading Frame; Ras, Rat Sarcoma; PTEN, Phosphatase and tensin homolog; PDGFA, Platelet Derived Growth Factor Alpha; Wnt, Wingless-related integration site; BRAF, Rapidly Accelerated Sarcoma B; MAPK, Mitogen Activated Protein Kinase; ALK, Anaplastic Lymphoma Kinase; RON, Recepteur d’Origine Nantais; MSP, Macrophage Stimulating Protein; APC, Adenomatous Polyposis Coli; EGFR, Epidermal Growth Factor Receptor; FLT3, FMS-like Tyrosine Kinase 3; JAK2, Janus Kinase 2.

## 9. Molecular Prognostic Algorithm

**Figure 1:**
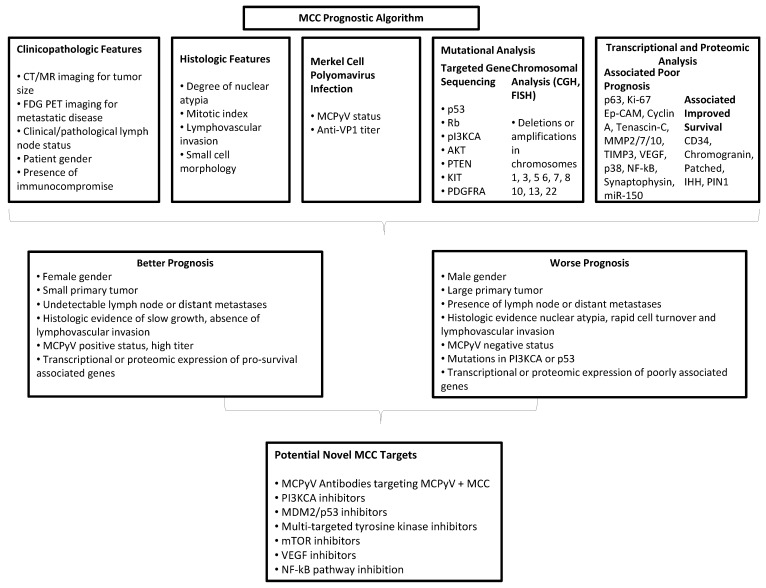
Prognostic algorithm for MCC.

CT, computer tomography; MR, magnetic resonance; MCPyV, Merkel cell polyomavirus; Rb, Retinoblastoma; PI3K, Phosphatidylinositol-4,5-bisphosphate 3-kinase; PTEN, PTEN, Phosphatase and tensin homolog; PDGFRA, Platelet Derived Growth Factor Receptor Alpha; Ep-CAM, Epithelial Cell Adhesion Molecule; IHH, Indian Hedgehog; MMP, matrix metal oproteinase; TIMP3, Tissue Inhibitor of Metalloproteinase 3; VEGF, Vascular Endothelial Growth Factor; PIN1, Peptidyl-prolyl cis-trans isomerase 1.

Although MCC prognosis is on average quite poor, there is considerable range in survival, yet we have limited capacity to predict outcome. Most studies on survival in MCC have historically focused on clinicopathologic features including tumor size and location, histologic features such as nuclear atypia and lymphovascular invasion, and metastases to lymph nodes and distant sites. However, with advances in molecular diagnostics, characterization of MCC signatures will provide better accuracy for predicting prognosis in the individual patient. There are already multiple studies correlating expression of one or several genes with prognosis in MCC, typically via protein expression using immunohistochemistry (IHC), and the information from these resources could be synthesized to create a prognostic molecular profile (Table 4). MCPyV status may also be an important bifurcation, as virus negative tumors have been shown to have worse prognosis in some series (Figure 1) [32,39].

**Table 4:** Markers associated with prognosis in MCC.

Expressed Marker	Association with MCC Prognosis
MCPyV	Associated with LT-Ag and RB1expression and absence of TP53 expression, and was associated with improved disease specific and overall survival (*p* < 0.01) on univariate analysis [95]. Polyomavirus-positive Merkel cell carcinomas showed better prognosis with one spontaneous regression case and significantly higher expression of retinoblastoma protein (*p* = 0.0003) and less TP53 expression (*p* = 0.0005) compared to MCPyV negative MCC [32].
Intratumoral CD8	Independent predictor of survival on multivariate analysis (*p* = 0.01) [31].
Anti-LTAg	Associated with MCPyV infection, titer level correlated with disease progression. Rise in T-Ag titer preceded tumor recurrence, may have biomarker potential [96].
Anti-VP1	High anti-VP1 titers associated with improved progression free survival in MCC patients (*p* = 0.003) [97].
p63	p63 is expressed in more advanced disease, though its role as a prognostic tool is undetermined. In two different series, p63 expression was significantly associated with decreased survival [98,99]. However, a separate study of 95 patients found no correlation between p63 and prognosis [100].
Ki-67	Ki-67 labeling index exceeding 50% correlated with poor prognosis [101].
Ep-CAM	Highly expressed in metastasizing MCC [102].
Cyclin A, Tenascin-C	Associated with worse prognosis [103].
Patched and IHH	Sonic Hedgehog (SHH) pathway proteins were frequently and intensely over-expressed in the MCCs in this study (Sonic hedgehog, 93%; Indian hedgehog, 84%; Patched, 86%; Smoothened, 79%; Gli-1, 79%; Gli-2, 79%; Gli-3, 86%) compared with control samples. High levels of Patched and Indian hedgehog were significantly associated with an increase in patients overall (*p* = 0.015) and recurrence-free survival (*p* = 0.011), respectively [104].
MMP2/7/10, TIMP3, VEGF, P38, NF-kappaB, and Synaptophysin	Expression correlated with metastatic tumor spread [105].
PIN1	Binds and stabilizes TP53, causing cell cycle arrest and growth inhibition. Found to be expressed in all 27 MCC samples studied to varying degrees. High expression was significantly associated with improved overall survival (50% 5-years survival *vs.* 14%; *p* = 0.03) [106].
miR-150	miR-150, mi-146, miR-630, miR-483-5p, and miR-142-3p associated with worse prognosis, though only miR-150 reached statistical significance [77].
CD34 and Chromogranin	Trend towards favorable prognosis [107].

MCPyV, Merkel cell; Rb, Retinoblastoma; Ep-CAM, Epithelial Cell Adhesion Molecule; IHH, Indian Hedgehog; MMP, matrix metalloproteinase; TIMP3, Tissue Inhibitor of Metalloproteinase 3; VEGF, Vascular Endothelial Growth Factor; PIN1, Peptidyl-prolyl cis-trans isomerase 1.

## 10. Conclusions

Despite a recent plethora of mutational studies in MCC, we have yet to find a disruptive pathway that is the driving factor in the majority of cases. Mutations in TP53, Rb, and PIK3CA, found in the minority of patients, may provide an avenue for the development of therapeutic targets for certain patients. However, as this review suggests, continued searches for gene mutations, which are only one facet of cancer pathophysiology, may be of limited value. Many of the oncologic pathways seen in other cancers have been rigorously evaluated for missense and nonsense mutations in MCC with disappointingly low yield. It is possible that we have merely been looking at the wrong pathways, and defining mutations are waiting to be discovered. However, as many of these studies have serendipitously found, although certain oncogenes are not mutated, they have abnormally high expression and activity that is likely still of clinical significance. MCC is elusive in that perhaps many of the driving mechanisms of this cancer are imbedded in still poorly understood processes such as immune surveillance, epigenetic alterations, aberrant protein expression, posttranslational modifications and microRNAs. Going forward, application of functional genomics and proteomics is greatly needed to provide the insights necessary to develop effective therapies.
